# Influence of Fillers and Ionic Liquids on the Crosslinking and Performance of Natural Rubber Biocomposites

**DOI:** 10.3390/polym13101656

**Published:** 2021-05-19

**Authors:** Magdalena Maciejewska, Anna Sowińska

**Affiliations:** Institute of Polymer and Dye Technology, Lodz University of Technology, Stefanowskiego Street 12/16, 90-924 Lodz, Poland

**Keywords:** ionic liquids, natural rubber, cellulose, hydrotalcite, silica

## Abstract

This work concerns the effect of fillers and ionic liquids on the cure characteristics of natural rubber (NR) compounds, as well as the mechanical and thermal properties of the vulcanizates. Three types of white filler were applied, such as cellulose, nanosized silica and hydrotalcite, to modify the performance of NR composites. Additionally, ionic liquids (ILs) with bromide anion and different cations, i.e., 1-butyl-3-methylimidazolium (Bmi) and 1-butyl-3-methylpyrrolidinium (Bmpyr), were used to improve the cure characteristics of NR compounds and functional properties of the vulcanizates. The type of filler and the structure of ILs were proved to affect the rheometric properties and cure characteristics of NR compounds as well as the performance of the NR vulcanizates. Owing to the adsorption of curatives onto the surface, silica reduced the activity of the crosslinking system, prolonging the optimal vulcanization time of NR compounds and reducing the crosslinking degree of the elastomer. However, silica-filled NR exhibited the highest thermal stability. Hydrotalcite increased the crosslink density and, consequently, the mechanical properties of the vulcanizates, but deteriorated their thermal stability. ILs beneficially influenced the cure characteristics of NR compounds, as well as the crosslink density and mechanical performance of the vulcanizates, particularly those filled with silica. Cellulose did not significantly affect the vulcanization of NR compounds and crosslink density of the vulcanizates compared to the unfilled elastomer, but deteriorated their tensile strength. On the other hand, cellulose improved the thermal stability and did not considerably alter the damping properties of the vulcanizates.

## 1. Introduction

Natural rubber (cis-1,4-polyisoprene) elastomer (NR) has been increasingly used, because it is renewable and environmentally friendly. The very good strength properties of NR composites determine their wide applications in the polymer technology for centuries [1]. However, apart from their rubber matrix properties, their crucial influence on the properties of elastomer composites is due to fillers, the composition of the curing system, the application of crosslinking coagents and other additives.

The incorporation of the filler into elastomer composites usually increases the modulus due to the inclusion of rigid particles in the soft elastomeric matrix. Moreover, the use of fillers considerably increases the tear resistance and abrasion resistance due to the filler’s reinforcing effect. The interactions between the filler particles are also crucial, leading to the formation of a filler network in the crosslinked elastomer matrix, which consequently affects the mechanical properties of the vulcanizates in both static and dynamic conditions. No less important are the filler–elastomer interactions, which can alter the structure of the final elastomeric network, leading to additional crosslinks [2]. Fillers can also modify other properties of rubber composites, such as thermal and dimensional stability [3]. On the other hand, fillers, especially silica, were reported to affect the efficiency of the crosslinking system and, consequently, the curing characteristics of the rubber compounds [3,4,5]. The most popular fillers of elastomer composites are carbon black and silica; however, fillers of natural origin, such as clays and cellulose, are increasingly used, especially in biocomposites.

Layered double hydroxides (LDHs) are known as anionic clays, and minerals with positively charged layers interleaved with ions and water molecules, to preserve the neutrality of the charge. Anionic clays generally do not occur in nature, while hydrotalcite (HTA), which is a natural anionic clay with the structural formula Mg_6_A_l2_(OH)_16_CO_3_ × 4H_2_O, was found to be naturally occurring mineral. Moreover, it has attracted much attention, especially in the development of new environmentally friendly catalysts. LDHs have been widely applied as adsorbents, catalysts, anion exchange materials and fillers in nanocomposites [6,7]. Their ability to exchange the anions make LDHs a unique class of layered solids that can be used as fillers bearing negative charge. LDHs can be mixed with the polymer matrices, converting their hydrophilic surfaces into hydrophobic ones [8]. It was also established that LDHs are characterized by a unique advantage over layered silicates, caused by their tunable structural homogeneity [9]. Currently, owing to the good properties of LDHs, such as ease of synthesis, lamellar structure and easily controllable particle size, high bound water content, non-toxicity, or high reactivity toward anionic organic species, these materials have attracted much attention, especially in the field of elastomer composites [10]. Applications of pure and modified LDHs have been widely studied in elastomer composites based on ethylene-propylene-diene rubber (EPDM) [11,12], chloroprene rubber (CR) [13], acrylonitrile-butadiene rubber (NBR) [14,15], carboxylated nitrile rubber (XNBR) [16,17], silicone rubber (SR) [18,19], polyurethane elastomers (PU) [20], solution styrene-butadiene rubber (S-SBR) [21] and epoxidized natural rubber (ENR) [22]. For example, Das et al. applied zinc-stearate-modified LDH as a mineral filler to ensure a more environmentally friendly rubber composites as compared to commercial formulations [23]. Due to the LDH’s modification, the rubber composites did not require the incorporation of ZnO and stearic acid in the sulfur curing system. The developed Zn-containing LDH nanoparticles provided zinc ions in the vulcanization process and have been used as highly anisotropic nanofillers to reinforce different rubber matrixes, e.g., NR, EPDM, NBR, XNBR and S-SBR. The use of Zn-containing LDH allowed for a significant reduction in Zn amount in vulcanizates. The content of zinc was approximately 10 times lower than in conventional rubber compounds containing ZnO. Moreover, introducing modified LDH improved the efficiency of crosslinking and resulted in transparent vulcanizates, which are difficult to achieve using ZnO in standard formulations. However, it should be noted that preparation of the zinc-stearate-modified LDH requires a chemical modification process. To our knowledge, this compound is not commercially available. On the other hand, Laskowska and co-authors [16] have reported the influence of HTA on the crosslinking reactions, mechanical and barrier properties of XNBR composites. HTA was proven to participate in the crosslinking of XNBR by the formation of ionic crosslinks, which resulted in strong and stiff elastomeric composites, which exhibited a better ability to dampen vibrations and improved mechanical properties compared to the unfilled sample. However, 20–30 phr of HTA was necessary to obtain a degree of crosslinking and mechanical properties similar to that of the ZnO-containing vulcanizate. Moreover, the optimal vulcanization time of HTA-containing rubber compounds was significantly longer compared to XNBR cured with ZnO, which is a disadvantage from a technological perspective. To enhance the cure characteristics and crosslinking degree of HTA-containing elastomer composites, ionic liquids (ILs) can be successfully used [3]. To our knowledge, in the literature, there is no information on application of HTA in the presence of ILs to improve the cure characteristics and performance of NR composites.

Cellulose-based materials are a subject of research owing to their good processability, mechanical properties, high crystallinity, and renewable and biodegradable nature [24,25]. In recent years, synthetic fillers have been replaced by cellulosic nanofillers. However, the polar cellulosic nanofillers exhibit a high agglomeration ability in nonpolar elastomer matrices, which may seriously hinder their use in rubber nanocomposites [26]. Microcrystalline cellulose has been widely used in various industrial fields, i.e., the processing of food and plastics, pharmacy and perfumery. Microcrystalline cellulose was also reported to enhance the barrier, mechanical and thermal properties of plastics, polymer latexes or rubber composites, which resulted from the chemical composition of lignocellulosic fillers and their particle size [27,28,29]. For example, AlMaadeed et al. reported that cellulose was responsible for the high increase in the mechanical and thermal stability of low-density polyethylene composites [30]. On the other hand, Ni’mah et al. studied the influence of particle size and crystallinity of cellulose on the thermal and mechanical behavior of poly(L-lactic acid) acid (PLA) biocomposites [31]. An increase in the filler’s content resulted in a decrease in tensile strength, which was most likely due to the ability of cellulose particles to agglomerate in the PLA matrix. Although cellulose has been shown to be effective as a reinforcing filler for polymeric materials, achieving a homogeneous dispersion of its particles in the polymer composite and enhancing its compatibility with various polymer matrices is still a challenge. ILs have been widely reported as solvents, dispersing and dissolution agents of cellulose [32,33,34]. Moreover, ILs have been proven to facilitate filler dispersion [35,36], as well as to enhance filler–elastomer interactions [37] and, consequently, the mechanical properties of the vulcanizates [38,39] and thermal stability of elastomer composites. Therefore, it seems reasonable to use ILs in the NR composites filled with cellulose.

Elastomer compounds for potential applications such as the tire industry generally contain at least 30 phr of active fillers, i.e., carbon black or silica. Various types of silica are conventionally used as reinforcing fillers, since they improve the mechanical properties of elastomers, i.e., tensile strength, modulus, tear strength, heat build-up, flex fatigue, chunking resistance, as well as abrasion and skid resistance [40,41]. However, the addition of silica to rubber compounds has some disadvantages, which arise from weak elastomer–filler interaction and the incompatibility of silica with rubbers [42]. Due to the huge amount of silanol groups on its surface, which induce strong interparticle interactions via hydrogen bonding, silica shows a strong ability to agglomerate in the rubber matrix [43]. This results in poor dispersion and distribution of the filler in the elastomer matrix, and consequently deteriorates the mechanical properties of the vulcanizates [42]. Moreover, owing to a presence of functional groups, the surface of silica has a strong ability to adsorb the curing system, which reduces the efficiency of crosslinking and, consequently, the crosslink density of the vulcanizates [44,45,46]. Therefore, in order to reduce the agglomeration ability of silica particles and their ability to adsorb curatives, various modifications of silica surface have been performed [47,48,49] and various additives have been introduced to rubber compounds, e.g., silane coupling agents [50,51] and ILs [3,38,52,53]. In our previous work, the influence of ILs immobilized on the surface of different types of silica on the vulcanization, mechanical properties and thermal behavior of EPDM elastomer was established. The application of ILs-modified silica allowed the vulcanization of rubber compounds to be controlled without deterioration of the thermal stability, damping properties and resistance of the vulcanizates to thermo-oxidative aging. An improvement in the crosslink density and, consequently, in the tensile strength and hardness of the vulcanizates was observed due to the reduction in the silica surface’s ability to adsorb curatives [47]. Similar effect of ILs on the crosslink density was confirmed by Hussain et al. for silica-filled butadiene rubber [38]. The beneficial influence of the ionic liquid, i.e., 1-allyl-3-methylimidazolium chloride, on the curing characteristics was also demonstrated by Zhang et al. for the silica-filled NR nanocomposites [54]. However, most publications related to the application of ILs in NR composites focus on the use of ILs with different structures to improve the dispersion of carbon fillers and, hence, the conductivity and mechanical properties of rubber composites [55,56,57,58,59].

In this work, we focus on the effect of ILs and white fillers with different characteristics on the crosslinking and properties of NR biocomposites, including the thermal stability and mechanical performance under static and dynamic vulcanizate conditions. The aim of the research is to determine which of the tested fillers benefits from the use of ILs, and which ionic liquid cation structure is the most favorable for interaction with a particular filler and allows acceptable properties of the NR vulcanizates to be obtained.

## 2. Materials and Methods

### 2.1. Materials

**Scheme 1:**
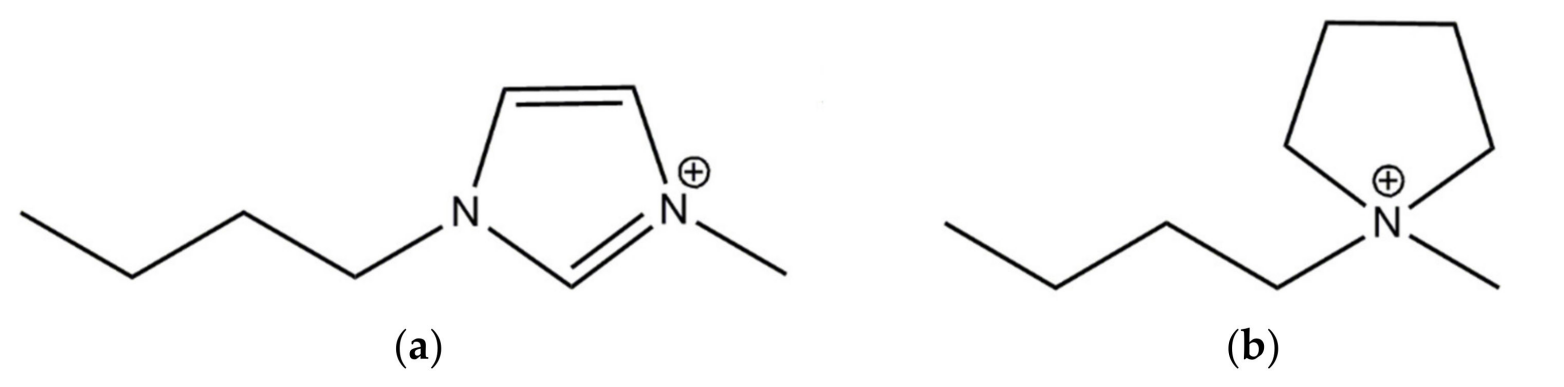
Structure of ILs cations: (**a**) 1-butyl-3-methylimidazolium; (**b**) 1-butyl-1-methylpyrrolidinium.

Natural rubber (NR, RSS1 type, *cis*-1,4-polyisoprene), with a density of 0.93–0.988 g/cm^3^ was purchased from Torimex Chemicals, Lodz, Poland. Three fillers with different characteristics were used, i.e., synthetic hydrotalcite (HTA, Sigma-Aldrich, Darmstadt, Germany), powdered cellulose (ARBOCEL^®^ CE 2910 HE 50 LV, JRS GmbH, Rosenberg, Germany), and silica AEROSIL^®^ 380 (A380, Evonik Industries, Essen, Germany). A conventional curing system was applied, containing sulfur (Siarkopol, Tarnobrzeg, Poland) as a crosslinking agent, microsized zinc oxide (ZnO, Huta Będzin, Będzin, Poland) as a vulcanization activator and 2-mercaptobenzothiazole (MBT, Brenntag Polska, Kędzierzyn-Koźle, Poland) as an accelerator. Stearin obtained from Akzo Nobel (Amsterdam, The Netherlands) was used as the softener and filler-dispersing agent. Additionally, two ionic liquids (ILs) with characteristics given in Table 1 were applied (IoLiTec Ionic Liquids Technologies GmbH, Heilbronn, Germany). These ILs consist of bromide anion and different cations with butyl and methyl substituents. The structures of ILs cations are presented in Scheme 1.

### 2.2. Preparation and Characterization of NR Compounds

NR compounds were prepared using a laboratory two-roll mill (Bridge, Rochdale, UK) with the following rolls dimensions: D = 200 mm, L = 450 mm. The friction and the width of the gap between rollers were 1–1.2 and 1.5–3 mm, respectively, whereas the rotational speed of the front roll was 16 min^−1^. The average temperature of the rolls during compounding was approximately 30 °C. First, the reference unfilled rubber compound (R0) was prepared, and then three masterbatches containing an appropriate filler were manufactured. The general recipes of the reference NR compounds (R0–R3) are given in Table 2. Then, each masterbatch was divided into three equal pieces and the vulcanization activator (ZnO) previously mixed with a proper IL was added to two of these pieces (the rubber compounds were marked as IL1-IL6). In contrast, only ZnO without IL was introduced into the third piece (R1–R3). The general formulations of NR compounds containing ILs are presented in Table 3.

Rheometric measurements were performed according to the ISO 6502 [60] standard procedures using the rotorless D-RPA 3000 rheometer (MonTech, Buchen, Germany) at 160 °C. The measurements helped determine: the optimal vulcanization time (t_90_), which is the time needed for rheometric torque to gain 90% of the maximum attainable torque value (S_max_), the scorch time (t_02_) and the torque increase (∆S) during vulcanization, which is described by Equation (1)

(1) $$\Delta S=S_{\max}-S_{\min\;}$$

where S_min_ is the minimum of the torque during rheometric measurement (dNm). Using the same analyzer, the viscosity of uncured rubber compounds was determined at 100 °C as a function of the time. The time of measurement was 30 min and the frequency was 1.667 Hz. The vulcanization of NR compounds was carried out at 160 °C, using a hydraulic press with electrical heating.

Differential scanning calorimeter DSC1 (Mettler Toledo, Greifensee, Switzerland) was used to study the effect of fillers and ILs on the temperature and enthalpy of NR crosslinking. Using a STAR^e^ software, the onset temperature of the peak corresponding the crosslinking reactions was determined, following the procedure given in the ISO 11357-1 [61] standard. The measurements were carried out for small pieces of rubber compounds, with a mass of approximately 10 mg, which were heated from −150 °C to 250 °C, with a heating rate of 10 °C/min. Liquid nitrogen was applied for sample-cooling, and the protective gas used during measurements was nitrogen, with the flow rate of 80 mL/min.

The density of crosslinks in the elastomer network was examined according to the ISO 1817 [62] standard, based on the equilibrium swelling of the vulcanizates in toluene, which was performed for 48 h at room temperature. Then, the Flory–Rehner Equation (2) [63] was applied to calculate the crosslink density of the vulcanizates

(2) $$\nu_{t}=\frac{\ln\left(1-V_{r}\right)+V_{r}+{\mu V}_{r}^{2}}{V_{0}\left(V_{r}^{\frac{1}{3}}-\frac{V_{r}}{2}\right)}$$

where ν_t_ is the crosslink density of vulcanizates (mole/cm^3^); V_0_, the molecular volume of solvent (106.7 cm^3^/mole); χ, the Huggins parameter of the NR elastomer–toluene interaction, given by Equation (3) [64], where V_r_ is the volume fraction of elastomer in swollen gel.

(3) $$\chi =0.780+0.404V_{r}$$

Fourier transform infrared spectroscopy (FT-IR) absorbance spectra were recorded in the range of wavenumbers from 4000 to 400 cm^−1^ with 64 scans. The measurements were performed with the use of a Thermo Scientific Nicolet 6700 FT-IR (Thermo Fisher Scientific, Waltham, MA, USA) spectrometer with OMNIC 3.2 software. Attenuated Total Reflectance (ATR) equipped with a single reflection diamond ATR crystal was used for all investigations.

Zwick Roell 1435 (Ulm, Germany) universal testing machine was applied to examine the mechanical properties of vulcanizates. Tensile tests were carried out in static conditions according to the ISO 37 [65] standard procedure for five dumb-bell-shaped samples with a thickness of approximately 1 mm, and a measuring section width of 4 mm. The crosshead speed during tensile tests was 500 mm/min.

Thermogravimetric analysis (TG) was performed to study the thermal stability of fillers, ILs and finally NR vulcanizates. Thermogravimetry/differential scanning calorimetry TGA/DSC1 analyzer (Mettler Toledo, Greifensee, Switzerland) was employed to conduct measurements for the samples with a mass of approximately 10 mg placed in the open alumina crucibles. TG analysis of pure fillers and ILs was carried out in the temperature range of 25–500 °C in argon atmosphere (50 mL/min), with a heating rate of 10 °C/min, whereas TG measurements for NR vulcanizates were conducted using a two-step procedure. First, small pieces of the vulcanizates were heated in the temperature range of 25–600 °C in an inert gas (argon, 50 mL/min). Next, the gas was changed into air (50 mL/min), and samples were heated to 900 °C with the same heating rate.

Dynamic mechanical analysis (DMA) was carried out in tension mode using a DMA/SDTA861e analyzer (Mettler Toledo, Greifensee, Switzerland). The measurements were performed in the temperature range of −150–60 °C with a heating rate of 3 °C/min, a frequency of 1 Hz, and a strain amplitude of 4 µm. Liquid nitrogen was used as a cooling medium. The specimens had a strip shape with a width of 4 mm, length of 10.5 mm and a thickness of approximately 1 mm.

Scanning electron microscopy (SEM) images of the vulcanizate containing silica and BmiBr were taken using a HITACHI S-4700 (Hitachi, Mannheim, Germany) microscope. Prior to the measurements, the fracture of the vulcanizate was coated with a thin layer of carbon and analyzed. Energy-dispersive-X-ray spectroscopy (EDS) was used to study the distribution of silica, sulfur, zinc oxide and ionic liquid in the elastomer matrix.

## 3. Results and Discussion

### 3.1. Thermal Stability of Fillers and Ionic Liquids

**Figure 1:**
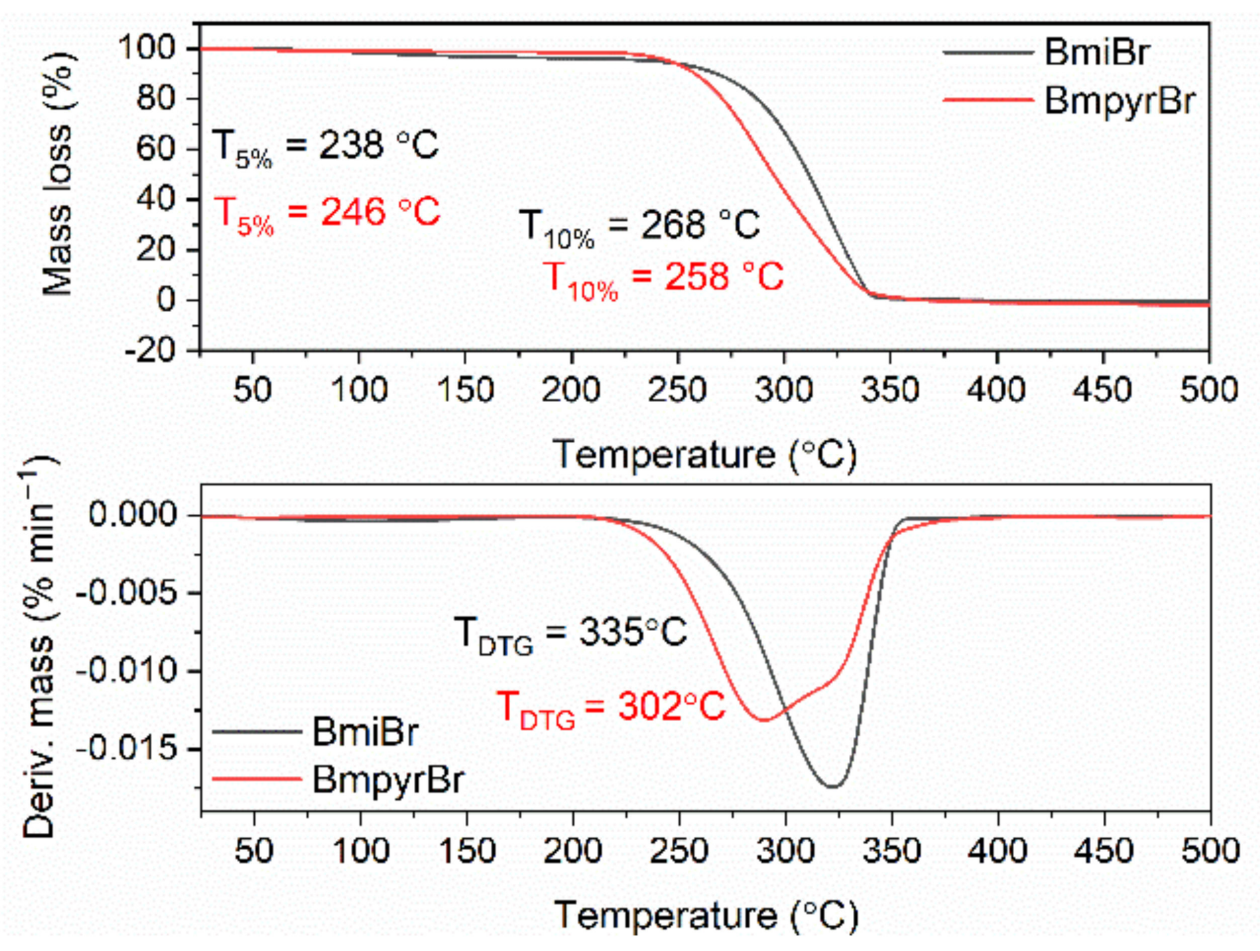
Thermogravimetric (TG) and derivative thermogravimetric (DTG) curves of BmiBr and BmpyrBr.

In the first step of studies, thermogravimetric analysis (TG) was employed to examine the thermal stability of pure ILs and fillers. The onset decomposition temperature (T_5%_) was determined as the temperature at 5% mass loss in relation to the initial mass of the sample. TG and derivative thermogravimetric (DTG) curves for pure ILs are presented in Figure 1.

**Figure 2:**
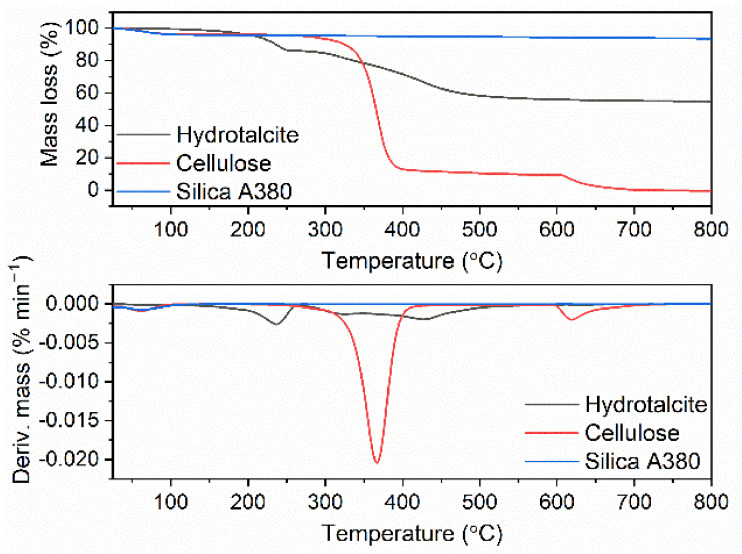
TG and DTG curves of pure fillers.

The thermogravimetric (TG) and derivative thermogravimetric (DTG) curves of the studied ILs with bromide anion were shown in Figure 1. Both ILs completely decomposed in the temperature range of 25–400 °C. Taking the T_5%_ into account, i.e., the onset decomposition temperature, slightly lower degradation temperature was exhibited by the imidazolium ionic liquid (BmiBr), (T_5%_ of approximately 238 °C) as compared to the pyrrolidinium one, i.e., BmpyrBr (T_5%_ of approximately 246 °C). However, the temperature at 10% mass loss (T_10%_) was approximately 10 °C higher for BmiBr than for BmpyrBr. Moreover, analyzing the TG and DTG curves, it was observed that the main stage of BmiBr thermal decomposition began at a higher temperature compared to that of BmpyrBr. Furthermore, the temperature of the peak in the DTG curve (T_DTG_) for BmiBr was approximately 33 °C higher than for BmpyrBr, so BmiBr showed a considerably higher temperature of the maximum mass loss rate compared to BmpyrBr, which proved to have a higher thermal stability of imidazolium ring of BmiBr than the pyrrolidinium ring of BmpyrBr. This is in good agreement with the data reported by Xue et al., which confirmed that imidazolium ILs are generally more thermally stable than pyrrolidinium salts [66]. An important aspect is the mechanism of ILs degradation, which depends on the anion and the structure of the ILs cation [67,68,69]. The bromide anion can attack the pyrrolidinium cation, which leads to the cleavage of C–N bond in the pyrrolidinium ring of the BmpyrBr and consequently in the lower T_10%_ and T_DTG_ temperature of BmpyrBr compared to that of BmiBr. Therefore, NR vulcanizates with BmiBr are expected to show a higher thermal stability than Bmpyr-containing vulcanizates. On the other hand, the thermal stability of elastomer composites also depends on the type of filler used. Hence, the thermal stability of pure fillers was investigated. The results are presented in Figure 2 and summarized in Table 4.

The investigated fillers demonstrated a completely different thermal behavior, which resulted from their different nature and composition (Figure 2). Silica A380 was the most thermally stable filler, which only lost the physically adsorbed water during the measurement. The mass loss corresponding to the water desorption was approximately 6.4%, while the peak temperature of the DTG curve related to this process was 70 °C. Similar silica behavior was reported by Maciejewska et al. [3] and Zhang et al. [70].

The most complex thermal decomposition was determined for cellulose (CE), which underwent three thermal transitions in the temperature range of 25–700 °C. The three-step thermal decomposition of CE was also reported by Scheirs et al. [71]. The first mass loss of approximately 10% occurred in the temperature range of 25–150 °C, with a T_DTG_ of approximately 70 °C. This was due to the water removal (dehydration) [71]. The second, and at the same time, the main stage of thermal decomposition of CE proceeded in the temperature range of 150–500 °C, with a T_DTG_ temperature of 365 °C and the mass loss of 86.5%. In this step, cellulose, which is a polysaccharide may undergo chemical and physical reactions such as hydrolysis, decarboxylation, depolymerization, and a sugar-based conversion to form volatile substances, such as H_2_, CH_4_, CO, and CO_2_ [72]. According to Banyasz et al., CE during pyrolysis can undergo the reaction of transglycosylation, producing a volatile tar consisting of levoglucosan, or generate gases (hydroxyacetaldehyde, formaldehyde, and CO) via another intermediate [73,74]. Additionally, the reaction of transglycosylation can lead to the dimer and trimer, such as cellobiosan [75] and cellotriosan [76], respectively, which are components of non-volatile tar. However, their volatilization is possible and may proceed after additional fragmentation with the evolution of light gases. A detailed description of CE’s thermal decomposition was presented by Mamleev et al. [77]. The third mass loss of approximately 6% was observed at a temperature above 550 °C with a T_DTG_ of 630 °C. The CE filler completely decomposed wiht the formation of gas products under the measurement conditions. The pyrolysis of CE into gas products was confirmed by Banyasz et al. [73,74].

The thermal decomposition of synthetic hydrotalcite (HTA), which is a magnesium aluminum hydroxycarbonate (Mg_6_Al_2_(CO_3_)(OH)_16_·4H_2_O), was a two-step process. The first mass loss was approximately 14% and occurred at a temperature below 250 °C with a T_DTG_ of 240 °C. It resulted from the desorption of water that was physically adsorbed by the HTA, accompanied by the dehydration (removal of water from hydrates). In the second stage, the dehydroxylation occurred together with decarbonation, i.e., removal of CO_2_ [78], in the temperature range of 250–800 °C, with a T_DTG_ of approximately 445 °C and a mass loss of 31.3%. The total mass loss resulting from HTA thermal decomposition was 45.2%.

### 3.2. Effect of Fillers and Ionic Liquids on Cure Characteristics of NR Compounds and Crosslink Density of Vulcanizates

The rheometric properties of NR compounds were investigated to determine the effect of the applied fillers and ILs on the curing parameters. The measurements were performed at 160 °C and the results are summarized in Table 5.

Analyzing the obtained results, it could be concluded that the type of filler and ILs affected the cure characteristics of NR compounds. Regarding the unfilled rubber compounds, the application of ILs, i.e., BmiBr and BmpyrBr, did not have a considerable effect on the minimum rheometric torque. However, the maximum torque and, consequently, the torque increase in NR compounds containing BmiBr and BmpyrBr were slightly higher compared to the unfilled sample, which suggests a slightly higher crosslinking degree of the elastomer containing ILs. On the other hand, the ILs did not significantly alter the scorch time and optimal vulcanization time compared to the unfilled NR.HTA, and CE did not have a significant influence on the minimum torque and the scorch time of the NR compounds compared to the unfilled rubber. A similar effect to these fillers was observed for the optimal vulcanization time (t_90_), which was approximately 2 min for both the unfilled NR and rubber compounds filled with HTA or CE. The longest scorch time and optimal vulcanization time were determined for the A380-filled rubber compound (approximately 4 min and 16 min, respectively) owing to the adsorption of curatives onto the silica surface, which decreased the activity of the crosslinking system. The same influence of silica A380 on the t_90_ was observed by Maciejewska et al. for NBR [3] and SBR compounds [79]. Moreover, silica A380 significantly increased the S_min_, so the viscosity of the uncrosslinked rubber compound compared to the unfilled NR. All of the used fillers enhanced the maximum torque during vulcanization in comparison to the unfilled NR composite. This was due to the introduction of the rigid phase of the filler into the soft elastomer matrix. A380-filled NR compounds exhibited a significantly higher S_max_ compared to the rubber compounds with HTA and CE. This could be attributed to the hydrodynamic effect of the silica, as well as to filler–filler and filler–polymer interactions [2,80].

**Figure 3:**
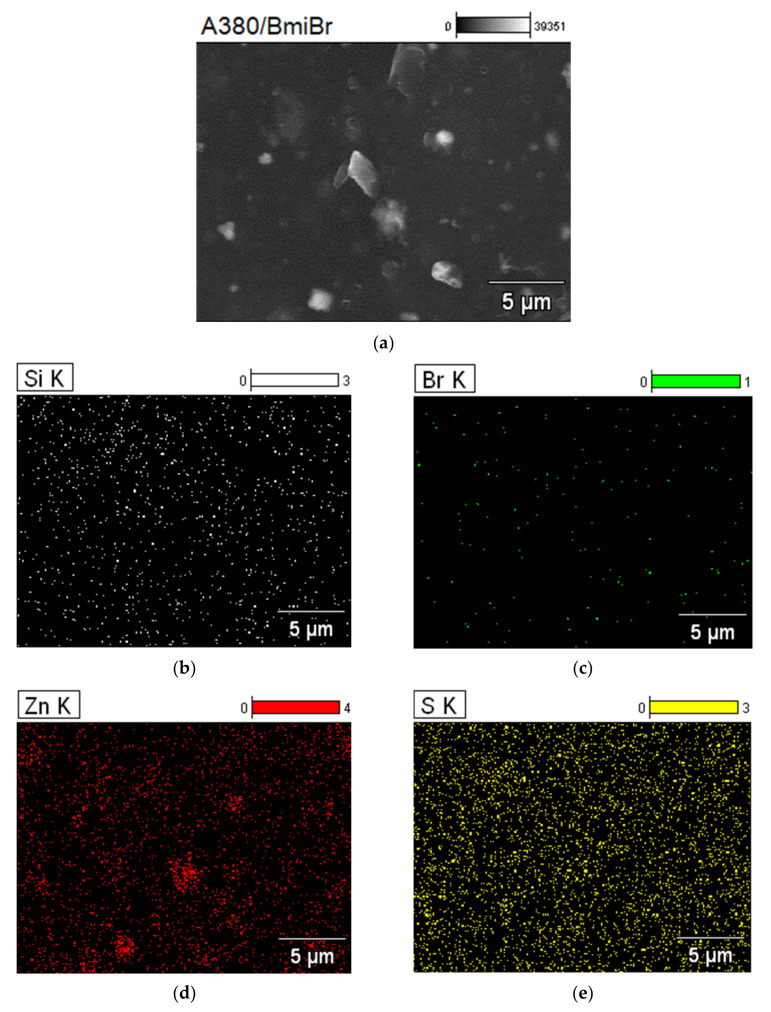
Scanning electron microscopy (SEM) image and Energy-dispersive X-ray spectroscopy (EDS) maps for A380-filled vulcanizate containing BmiBr: (**a**) SEM image; (**b**) EDS map for Si; (**c**) EDS map for Br; (**d**) EDS map for S; (**e**) EDS map for Zn.

In the case of NR compounds filled with CE or HTA, the addition of ILs did not significantly affect the values of S_min_, t_02_ and t_90_. Regarding S_max_ and, consequently, ΔS, BmiBr slightly increased these parameters, especially for HTA-filled NR compounds, whereas BmpyrBr did not significantly affect them in comparison with the values characteristic of NR compounds without ILs. On the other hand, ILs, regardless on their cation, had a beneficial influence on the cure characteristics of NR filled with silica A380. ILs reduced the S_min_ and, therefore, the viscosity of the uncrosslinked rubber compounds, which facilitated their processing. Moreover, the addition of ILs to NR compounds filled with silica caused a considerable increase in the S_max_ and, consequently, ΔS, in comparison to NR compounds without ILs. This was due to the improvement in elastomer crosslinking degree. The positive impact of ILs on the vulcanization of NR compounds containing silica also resulted in a reduction in the t_90_ from 16 min to approximately 2 min, which is very important for technological and economic reasons. The beneficial influence of ILs on the cure characteristics of A380-filled NR compounds was probably due to the adsorption of ILs on the silica surface, which limited its ability to adsorb the crosslinking system, and consequently improved the crosslinking efficiency. This should also enhance the crosslink density of the vulcanizates. To confirm this assumption, SEM/EDS analysis was performed for the A380-filled vulcanizate containing BmiBr. SEM images with corresponding EDS maps are presented in Figure 3. Analysis of the SEM image and EDS maps revealed that silica nanoparticles were quite homogeneously dispersed in the elastomer matrix (Figure 3b), similarly to the bromide anion of BmiBr (Figure 3c). Regarding the distribution of the curing system, a uniform dispersion of sulfur could be seen (Figure 3d), whereas ZnO particles formed agglomerates which were several micrometers in size (Figure 3e). The adsorption of ILs on the silica surface was confirmed by Lei et al. for the silica-filled SBR composites [81]. Furthermore, the beneficial impact of ILs on the vulcanization was also reported for silica-filled NBR and SBR compounds [3,79].

**Figure 4:**
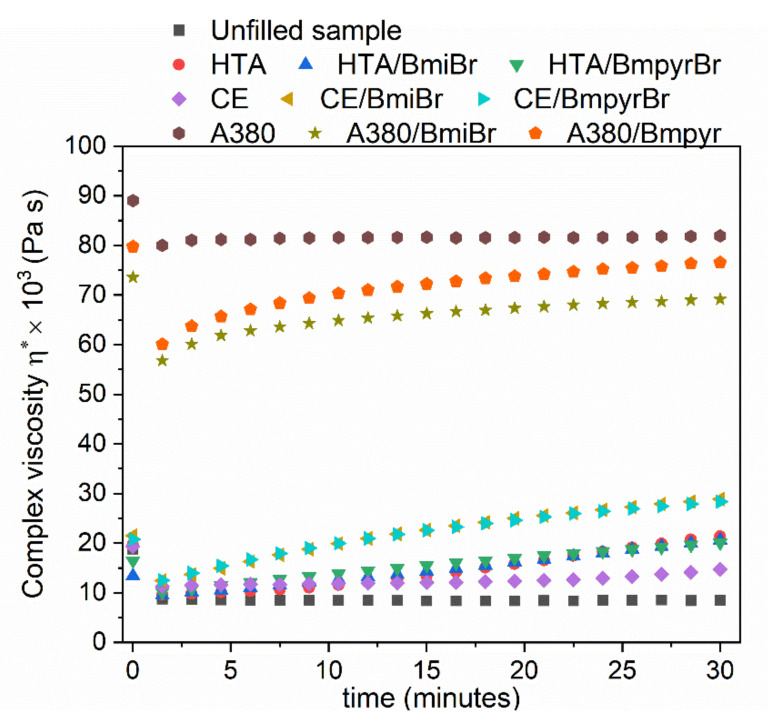
Effect of fillers and ILs on the complex dynamic viscosity (η*) of NR compounds as function of time.

It is commonly known that fillers affect the characteristics of rubber compounds processing. Uncured rubber behaves like a viscoelastic fluid during mixing and processing [82]. The viscosity is an important parameter of the rheological behavior of filled elastomer compounds. Rheometric measurements revealed that silica A380 significantly affected the minimum torque, and thus viscosity of the uncured NR compounds. Therefore, we decided to study the effect of fillers on the complex dynamic viscosity of NR compounds at 100 °C, a temperature similar to the processing temperature. Measurements were performed as a function of time to determine whether NR compounds, especially those with high viscosity, i.e., those containing silica, could undergo scorching or vulcanization at 100 °C. The results are presented in Figure 4.

The curves presented in Figure 4 illustrate the changes in the complex dynamic viscosity (η*) of NR compounds for the same shear rate (constant values of frequency and strain) over time. As expected, the addition of fillers and the type of filler used had a significant effect on the viscosity of NR compounds at 100 °C and their tendency to scorch at this temperature. Obviously, the unfilled NR compound was characterized by the lowest η* at 100 °C. Furthermore, the viscosity of the unfilled NR did not change over 30 min of heating, so this rubber compound had no tendency to scorch at 100 °C and can be safely processed at this temperature. HTA and CE slightly enhanced the viscosity of rubber compounds compared to the unfilled NR. The viscosity of the CE-filled rubber compound at 100 °C did not change significantly as a function of time, so this rubber compound exhibited a very small ability to scorch during processing at this temperature. The complex viscosity of HTA-containing NR compounds, especially those with ILs, increased slowly and slightly as a function of time, so these rubber are prone to scorching during processing at 100 °C. As expected, rubber compounds filled with silica A380 were characterized with the highest values of η* compared to other NR compounds. However, the viscosity of A380-containing NR without ILs did not change during heating at 100 °C for 30 min, so no scorching was observed during this time. ILs caused a considerable reduction in the viscosity of the uncured A380-filled NR compounds, probably due to the plasticizing effect of ILs. A slight increase in the viscosity of ILs-containing NR compounds was observed as a function of time. Thus, it was concluded that ILs-containing NR compounds filled with silica are more susceptible to scorching during processing at 100 °C than those without ILs. A similar influence of ILs on the scorching ability was achieved for rubber compounds filled with CE.

**Figure 5:**
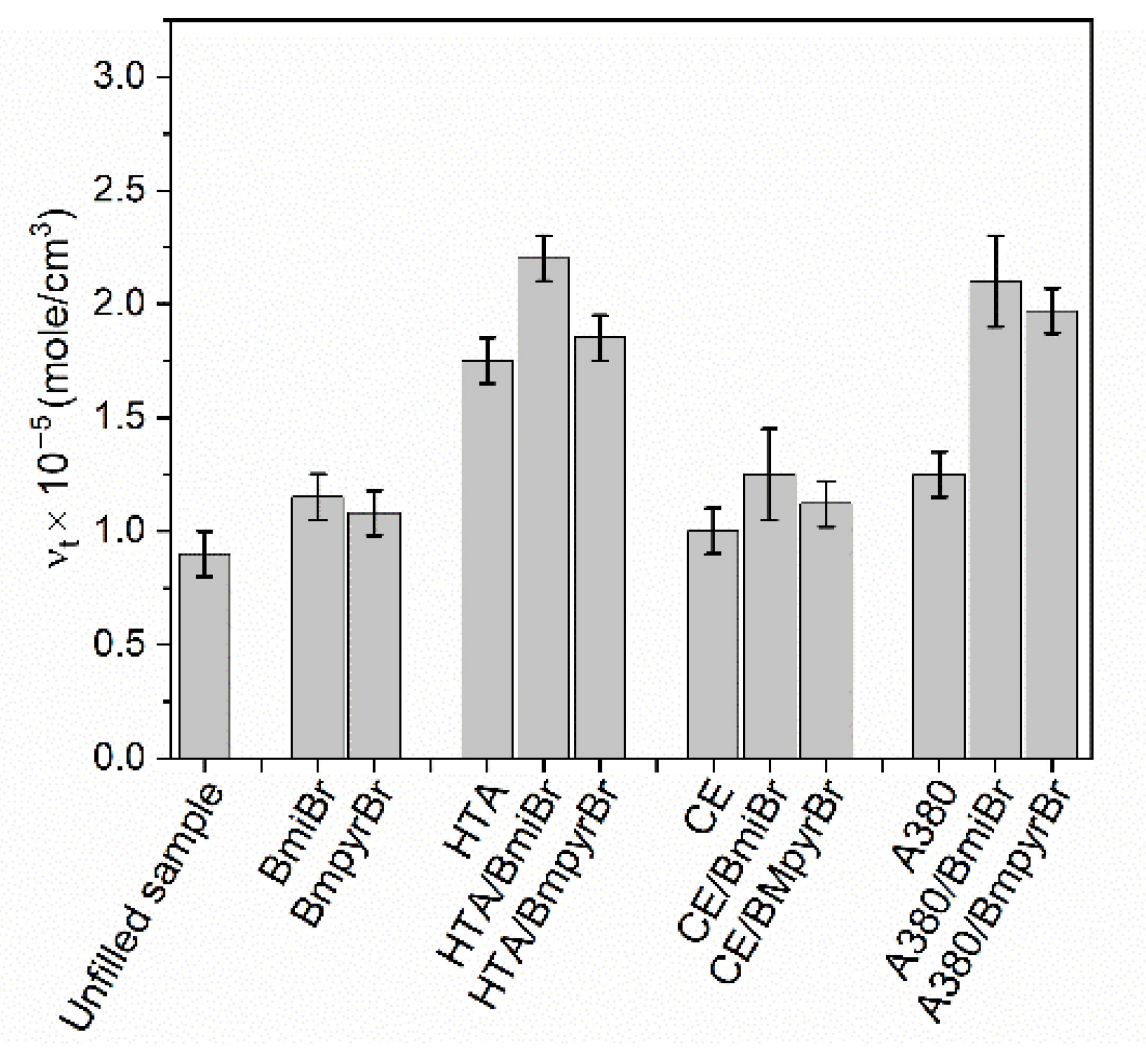
Effect of fillers and ILs on the crosslink density of NR vulcanizates.

Having investigated the cure characteristics of NR compounds, in the next step of the research, the equilibrium swelling method was used to examine the effect of fillers and ILs on the crosslink density of NR vulcanizates. The results are shown in Figure 5.

The application of a conventional crosslinking system containing sulfur results in sulfur crosslinks between elastomer chains. The spatial structure of the vulcanizates that is created depends on different factors, such as the type of elastomer and crosslinking agent, and the presence of other ingredients, including fillers, which can cause the chemical bonding between their surface functional groups and the rubber matrix. No less important are physical interactions between the filler and the polymer matrix. These connections may act as additional physical network nodes, affecting the density of crosslinks in the final elastomer network [83]. Therefore, the presence of investigated fillers and ILs affected the crosslink density of the vulcanizates, causing it to increase in comparison with the unfilled vulcanizate. However, in the case of vulcanizates filled with CE and A380, only a slight increase in the crosslink density was achieved due to the hydrophilic character of these fillers, resulting in their incompatibility and, consequently, poor interaction with the hydrophobic NR elastomer matrix [80]. On the other hand, HTA significantly increased the crosslink density compared to the unfilled NR vulcanizate. A similar effect of HTA on the vulcanizates’ crosslink density was reported for EPDM [11], NBR [3] and XNBR vulcanizates [16]. Owing to the Mg-Al-OH framework, HTA may provide metal ions for the crosslinking of carboxylated elastomers such as XNBR, or promote sulfur crosslinking by partially replacing the role of the conventional vulcanization activator, i.e., ZnO. HTA may also act as an additional activator of the sulfur vulcanization [10]. No less important is the alkaline character of HTA, since vulcanization proceeds more effectively in an alkaline environment [84].

The addition of ILs, such as BmiBr and BmpyrBr, to an unfilled NR compound caused a slight increase in the crosslink density of the vulcanizates without fillers, which is supposed to be due to the improved dispersion of the crosslinking system in the elastomer matrix and some catalytic effect of ILs in the interfacial crosslinking reactions, as postulated and confirmed by SEM images for the unfilled vulcanizates of an acrylonitrile–butadiene elastomer [85].

Regardless of the filler type, ILs increased the crosslink density of NR vulcanizates. Furthermore, vulcanizates with BmiBr exhibited slightly higher crosslink densities than those containing BmpyrBr. The beneficial influence of ILs on the crosslink density was especially evident for the vulcanizates filled with silica A380, which shows a strong ability to adsorb the crosslinking system. As mentioned previously, ILs similar to silane coupling agents can adsorb on the silica surface, as postulated in the literature [81], reducing its ability to adsorb curatives, and consequently increasing the efficiency of vulcanization.

**Figure 6:**
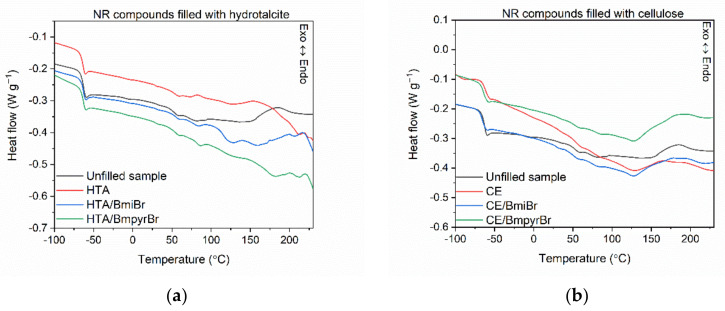

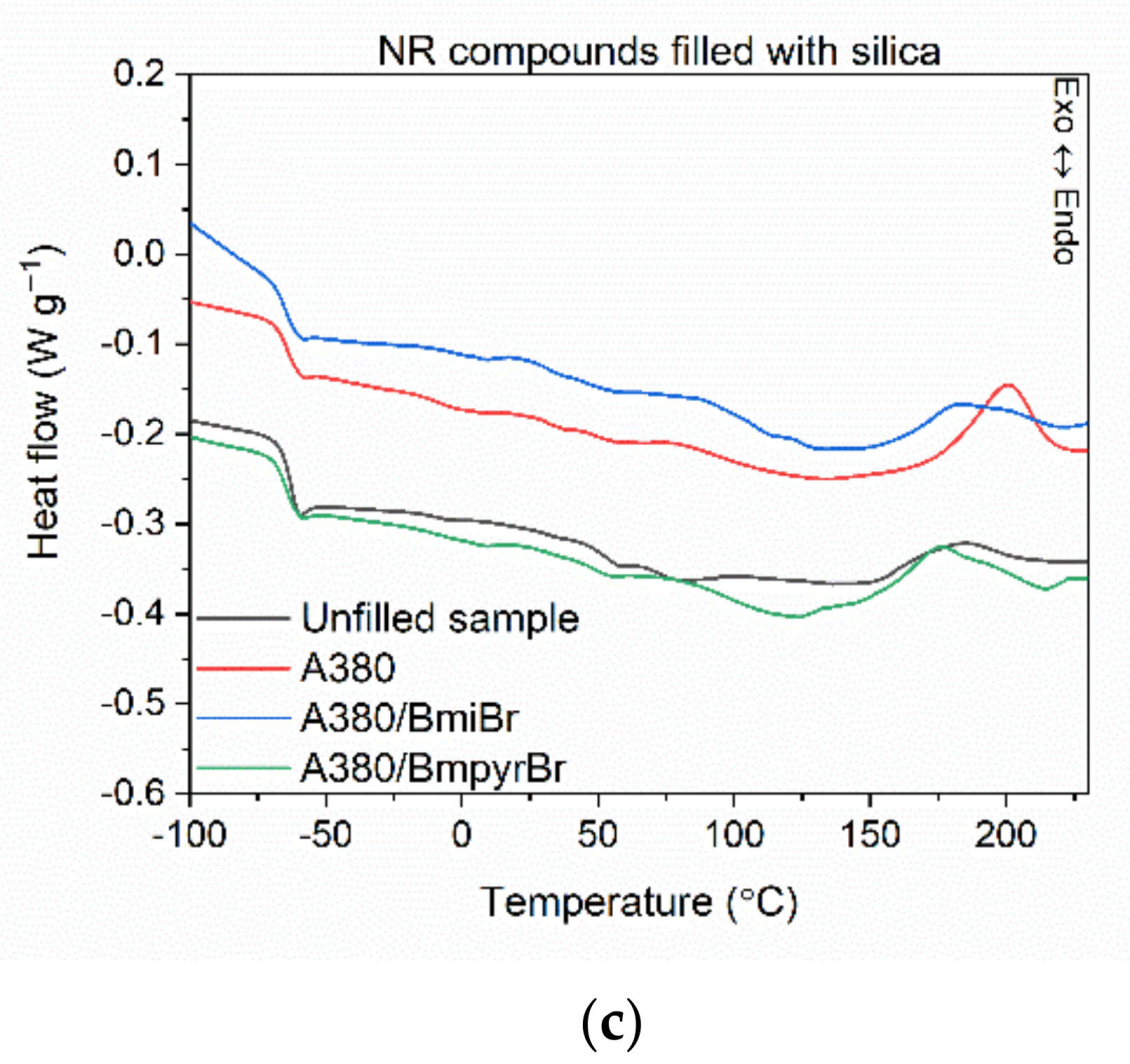
Differential scanning calorimetry (DSC) curves of NR compounds filled with: (**a**) HTA; (**b**) CE; (**c**) A380.

Having established the impact of fillers and ILs on the rheometric properties of NR compounds and crosslink density of the vulcanizates, we then investigated their effect on the temperature and enthalpy of vulcanization using DSC analysis. Additionally, the influence of fillers and ILs on the glass transition temperature (T_g_) of the NR elastomer was determined. The DSC curves of NR compounds are shown in Figure 6 and the results are summarized in Table 6.

Analyzing the results presented in Table 6, it was observed that introducing fillers and ILs into NR compounds did not have a significant influence on the glass transition temperature (T_g_); the differences in T_g_ were within the range of experimental error. The change in the heat capacity (∆C_p_) during the elastomer glass transition was slightly lower for filled NR compared to the unfilled sample, since fillers increase the stiffness of the composites.

Vulcanization of the unfilled rubber compound occurred in the temperature range of 147–215 °C with an enthalpy (ΔH) of 6.4 J/g. The type of filler significantly affected the temperature and enthalpy of the vulcanization. NR compounds filled with HTA and CE demonstrated an approximately 20 °C lower onset vulcanization temperature and considerably higher ΔH compared to the unfilled NR. Thus, the vulcanization of rubber compounds filled with HTA or CE began at a lower temperature and proceeded with the release of more heat (especially in the case of HTA), which may indicate a greater intensity of this process compared to the unfilled NR. On the other hand, silica A380 did not affect the onset vulcanization temperature as compared to the unfilled rubber (Figure 6c). The values of ΔH determined for A380-containing NR were higher than that of the unfilled rubber, but still lower compared to the rubber compounds filled with HTA or CE. Thus, DSC results confirmed the detrimental effect of silica A380 on the vulcanization of NR compounds as well as the beneficial influence of HTA, and the highest activity of this filler in the vulcanization. ILs had no significant effect on the onset vulcanization temperature of NR compounds, whereas the influence of ILs on the vulcanization enthalpy depended on the structure of the IL cation and the type of filler used. In the case of NR filled with HTA and CE, a positive effect of BmiBr on the ΔH was observed, while BmpyrBr did not considerably affect this parameter. On the other hand, ILs had no significant impact on the onset vulcanization temperature and ΔH of A380-filled NR (the values of ΔH were within the range of standard deviation), although rubber compounds with ILs vulcanized in the narrower range of temperature (146–219 °C) than the sample without ILs (149–232 °C).

### 3.3. Effect of Fillers and Ionic Liquids on Tensile Properties of NR Vulcanizates

The mechanism of stress transfer by the filler particles distributed in the elastomer matrix is one of the main factors determining the mechanical properties of elastomer composites [86]. Hence, the type and amount of added filler or the ability of a filler to create its own network in the elastomer matrix are significant. The crosslink density also has a considerable influence on the mechanical properties and hardness of the vulcanizates. The fillers and ILs used in this study significantly affected the number of crosslinks in the elastomer network compared to the unfilled vulcanizate. Therefore, it reasonable to examine their influence on the mechanical properties and hardness of the NR vulcanizates. The results are presented in Table 7.

From the data collected in Table 7, it follows that the changes in the SE_300_ modulus of the NR vulcanizates under the influence of fillers and ILs are consistent with the changes in the crosslink density. Therefore, vulcanizates with HTA and silica A380 exhibited a higher SE_300_ than the unfilled sample or vulcanizates containing CE. Moreover, the SE_300_ modulus of the vulcanizates containing ILs were significantly higher compared to the unfilled vulcanizate and those containing only fillers. As expected, the type of filler and the structure of ILs affected the tensile strength (TS) and elongation at break (EB) of NR vulcanizates, but the influence of the filler type was much more pronounced. The unfilled NR vulcanizate reached the TS value of 17.4 MPa and an EB of approximately 830%. Among the fillers used, only HTA showed the reinforcing effect towards NR elastomer. The HTA-filled vulcanizate without ILs exhibited approximately 3 MPa, higher TS than the unfilled sample, whereas vulcanizates containing CE and silica had a significantly lower TS (8.7 MPa and 9.1 MPa, respectively). The beneficial effect of HTA on TS may result not only from the reinforcing effect of this filler, but also from the highest crosslink density of the vulcanizates. Regardless of the filler used, the addition of ILs, especially BmiBr, increased the TS of the NR vulcanizates, probably due to the improvement in their crosslink density. The lowest TS was demonstrated by the silica-filled vulcanizate without ILs. This was due to the low crosslink density of this vulcanizate, as well as the high ability of silica to agglomerate in the elastomer matrix, resulting from strong filler–filler interactions. It should be noticed the ILs had the most significant influence on the TS of the A380-filled vulcanizates, improving it by approximately 3–5 MPa compared to the vulcanizate without ILs. This resulted from the greatest improvement in the crosslink density of the A380-filled vulcanizates caused by ILs. In addition, ILs similar to silanes could adsorb on the surface of silica, as postulated in the literature [81], preventing particles of this filler from agglomeration in the elastomer matrix. Regardless of the filler and IL applied, the NR vulcanizates exhibited an approximately 200–300% lower EB than the unfilled sample. This resulted from the increase in the stiffness of the vulcanizates due to the introduction of a filler into the elastomer matrix and, consequently, the reduction in the flexibility of the vulcanizates by the filler network. It was also due to the increase in the crosslink density of the vulcanizates compared to the unfilled one. The type of filler used and the structure of ILs had no significant influence on the EB of the vulcanizates.

The type of filler had a significant effect on the hardness of the vulcanizates. The unfilled vulcanizate exhibited the hardness of 30 ShA. All tested fillers enhanced the hardness of NR vulcanizates compared to the unfilled sample. As expected, the most pronounced influence on the hardness had a silica of A380, which increased the hardness to 49 ShA. ILs caused a further increase in the hardness of A380-filled vulcanizates due to the significant increase in their crosslink density. The HTA-filled vulcanizate showed the hardness of 37 ShA. ILs increase the hardness of HTA-containing NR by approximately 5 ShA compared to the unfilled sample. CE increased the hardness of NR vulcanizates to 42 ShA, but ILs did not significantly affect the hardness of CE-filled vulcanizates, as they also had no significant influence on the crosslink density compared to the sample without ILs.

### 3.4. Effect of Fillers and Ionic Liquids on Mechanical Properties of NR Vulcanizates under Dynamic Conditions

**Figure 7:**
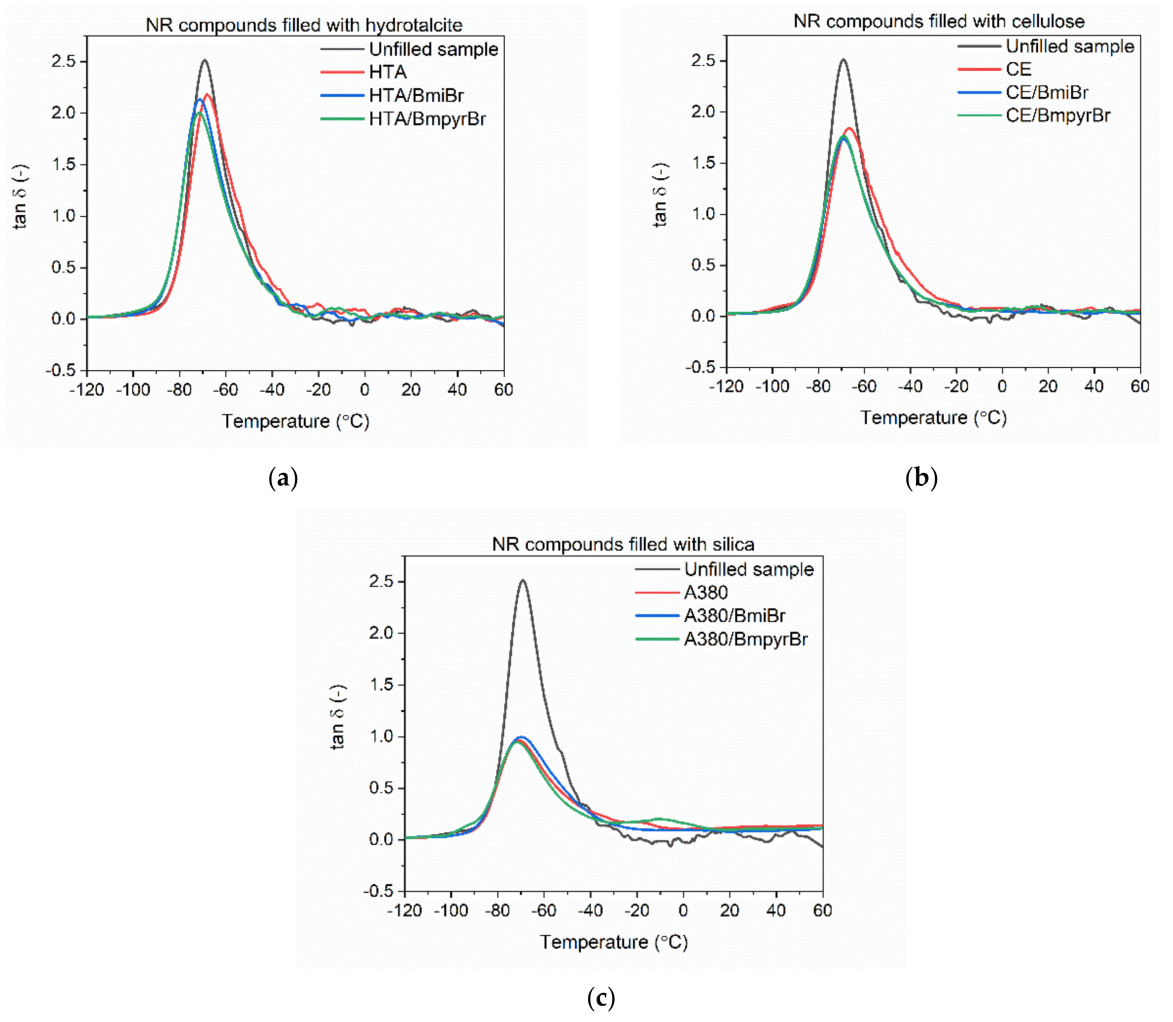
Loss factor (tan *δ*) curves versus temperature for NR vulcanizates filled with: (**a**) HTA; (**b**) CE; (**c**) A380.

Dynamic mechanical analysis (DMA) was used to study the effect of fillers and ILs on the viscoelastic properties of NR vulcanizates and their ability to dampen vibrations. The measurements were carried out as a function of temperature to determine the influence of the applied additives on the glass transition of NR elastomer and its properties in the rubbery elastic region. The results are summarized in Table 8, whereas the DMA curves of NR vulcanizates are plotted in Figure 7.

Regarding the effect of fillers and ILs on the glass transition temperature (T_g_), the results of DMA analysis correlate well with the data obtained by DSC. The type of filler and the ILs structure did not significantly affect the T_g_ of NR elastomer (Table 8). However, the T_g_ values determined by DMA were approximately 5 °C lower than those obtained from DSC measurements. This is due to the different conditions under which the T_g_ was determined using these two methods. During DSC measurements, a small piece of the vulcanizate was placed in the crucible and was only affected by the temperature, while, during the DMA analysis, the sample was subjected to both temperature and oscillating stretching with a given force, strain amplitude and frequency.

The loss factor is a measure of the material’s ability to dampen vibrations. Analyzing the DMA curves presented in Figure 7a–c, only one phase transition, i.e., the glass transition of NR elastomer, was observed in the tan δ curves of all vulcanizates in the measured temperature range. This transition is accompanied by a peak in the tan δ versus temperature curve, the maximum of which corresponds to the T_g_ of elastomer. Owing to the highest flexibility, the unfilled vulcanizate exhibited the highest mechanical loss factor at T_g_ (tan δ_Tg_ of approximately 2.5). The addition of fillers decreased the height of the tan δ peak due to the reduction in the chains’ mobility compared to the unfilled elastomer matrix [87]. As was concluded previously, based on the results of tensile properties, the applied fillers enhanced the stiffness and consequently reduced the flexibility of the NR vulcanizates and, therefore, the values of tan δ at T_g_. HTA had the least influence on the elastomer chains mobility and stiffness of NR vulcanizates, hence the values of tan δ at T_g_ decreased slightly as compared to the unfilled sample and were in the range of 2.0–2.2 (Figure 7a). CE had a greater impact on the mobility of elastomer matrix chains and stiffness of NR vulcanizates than HTA and, thus, the values of tan δ_Tg_ decreased by almost 30% in comparison with the unfilled sample (Figure 7b). As previously mentioned, silica A380 strongly stiffened the NR elastomer and significantly reduced the height of the tan δ peak. This revealed that the network formed by the silica particles severely limited the mobility of the chains in the elastomer matrix [86]. As a consequence, vulcanizates filled with silica demonstrated the tan δ at T_g_ of 1.0, and therefore were approximately 60% smaller compared to the unfilled vulcanizate (Figure 7c). Regardless of the filler used, ILs did not significantly affect the values of tan δ in the measured temperature range; therefore, ILs had no influence on the damping properties of the NR vulcanizates. Regarding the viscoelastic properties of the vulcanizates in the rubbery elastic region, HTA and CE did not alter the tan δ in the temperature range of 25–60 °C compared to the unfilled sample. The differences in tan δ values were within the range of measurement error and the DMA curves in this region almost overlapped. On the other hand, the A380-filled vulcanizates showed tan δ values more than twice as high as those of the unfilled vulcanizate in the rubbery elastic region. However, it resulted from the much higher stiffness of these vulcanizates and should not be interpreted as an improvement in the ability of the material to dampen vibrations compared to other vulcanizates.

### 3.5. Effect of Fillers and Ionic Liquids on Thermal Stability of NR Vulcanizates

**Figure 8:**
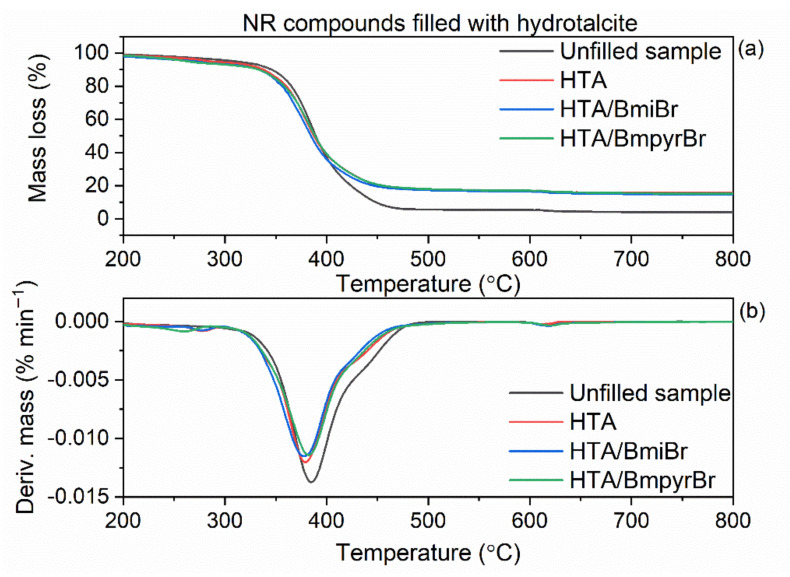
NR vulcanizates filled with HTA: (**a**) TG curves; (**b**) DTG curves.

**Figure 9:**
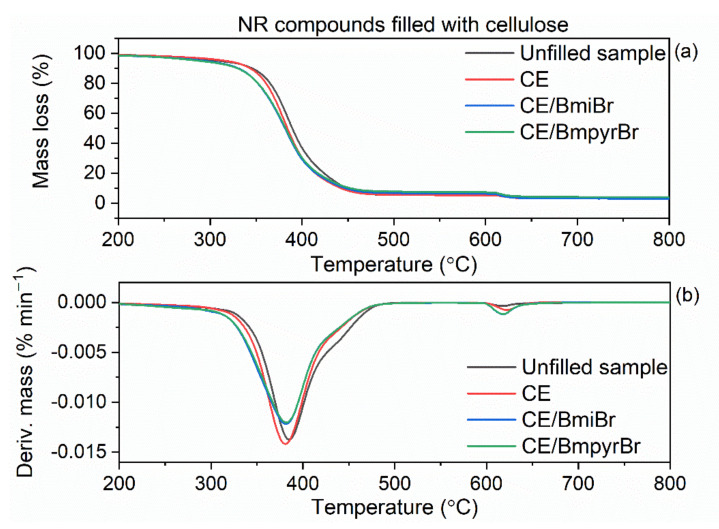
NR vulcanizates filled with CE: (**a**) TG curves; (**b**) DTG curves.

**Figure 10:**
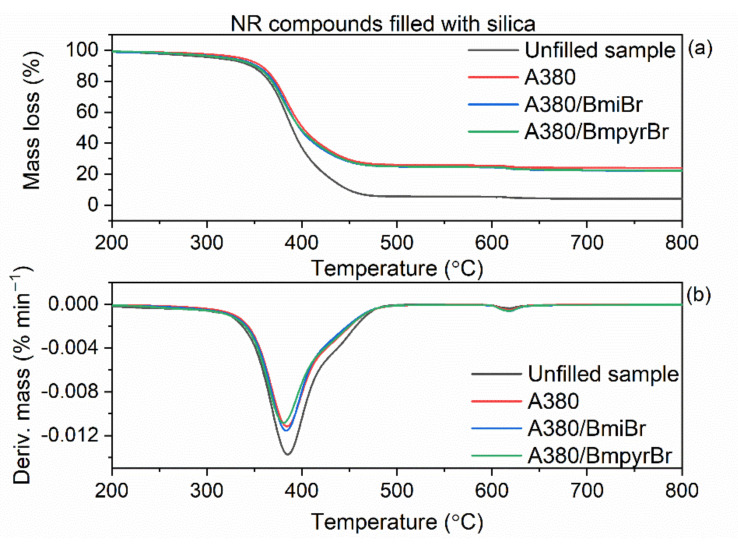
NR vulcanizates filled with silica A380: (**a**) TG curves; (**b**) DTG curves.

The ILs and fillers applied in this study were characterized by different levels of thermal stability, which may have an impact on the thermal stability of the NR vulcanizates. Therefore, TG analysis was performed for the vulcanizates to examine their thermal decomposition. The results are summarized in Table 9, while TG and DTG curves are presented in Figure 8, Figure 9 and Figure 10.

As expected, the type of filler and ILs significantly affected the onset decomposition temperature (T_5%_) of the vulcanizates, but had no considerable influence on the T_DTG_, or, therefore, the temperature of the maximum mass loss rate, which was in the range of 396–402 °C. Thermal decomposition of the unfilled vulcanizate started at a temperature of approximately 310 °C. Regarding the vulcanizates without ILs, the addition of CE and silica A380 improved the thermal stability of NR composites. It should be noted that the A380-filled vulcanizate exhibited a T_5%_ that was 25 °C higher than the unfilled sample. This was due to the high thermal stability of silica itself. In addition, the network formed by the silica nanoparticles, dispersed in the elastomer matrix, hindered the diffusion of gases and volatile products of thermal decomposition through the material, which resulted in improved thermal stability. Moreover, nanosized fumed silica was reported to act as a trap for polymeric char [88]. The beneficial effect of silica on the thermal stability of polymer composites was confirmed by Liu et al. [89] and Tarrío-Saavedra et al. [90], among others. On the other hand, HTA-filled vulcanizate showed a 26 °C lower T_5%_ compared to the unfilled sample. This resulted from the thermal decomposition of HTA, which started at a temperature above 150 °C.

Regardless of the filler used, ILs deteriorated the thermal stability of NR vulcanizates, especially those filled with CE (T_5%_ reduced by 23 °C and 29 °C for BmiBr and BmpyrBr, respectively). The least influence of ILs on T_5%_ was observed for the vulcanizates filled with silica A380. BmiBr reduced T_5%_ by 12 °C, whereas BmpyrBr was reduced by 15 °C, compared with the vulcanizate without ILs. Generally, vulcanizates containing BmiBr exhibited higher T_5%_ than that of the BmpyrBr-containing sample. This resulted from the higher thermal stability of BmiBr in comparison with BmpyrBr.

Analyzing the thermal decomposition of NR vulcanizates, the first mass loss determined in argon at 25–600 °C corresponds to the pyrolysis of rubber and thermal decomposition of the organic ingredients present in the vulcanizate, i.e., MBT accelerator, ILs and CE. In the case of HTA-filled vulcanizates, the first mass loss is also related to the decomposition of HTA, as confirmed by the TG results of the pure filler. The highest mass loss in the temperature range of 25–600 °C was achieved for the unfilled sample and for the vulcanizates containing CE. It should be noted that CE is an organic filler, which underwent significant thermal decomposition in this temperature range (Figure 2). The lowest mass loss in in the temperature range of 25–600 °C was observed for the vulcanizates filled with silica A380, since this filler is the most thermally stable of the tested fillers and only loses adsorbed water. The second mass loss occurred in air atmosphere at 600–800 °C. In this stage, the carbon residue after the pyrolysis of elastomer and organic additives was burnt, so this mass loss was the highest for the vulcanizates containing CE and ILs. As expected, all vulcanizates with ILs were characterized by a higher mass loss in this temperature range. The residue after thermal decomposition at 800 °C consisted of ash and mineral compounds; therefore, it was the highest for the vulcanizates filled with silica and HTA. For the A380-filled vulcanizates, the residue at 800 °C consisted of the filler and vulcanization activator, i.e., ZnO. For the unfilled sample and vulcanizates with CE, only ZnO remained after thermal decomposition, whereas, in the case of HTA-filled vulcanizates, it was ZnO, and some mineral residue containing mixed oxides of magnesium and aluminum. Most importantly, regardless of the filler used, the NR vulcanizates were thermally stable up to a temperature of approximately 300 °C.

### 3.6. FT-IR Analysis of NR Vulcanizates

**Figure 11:**
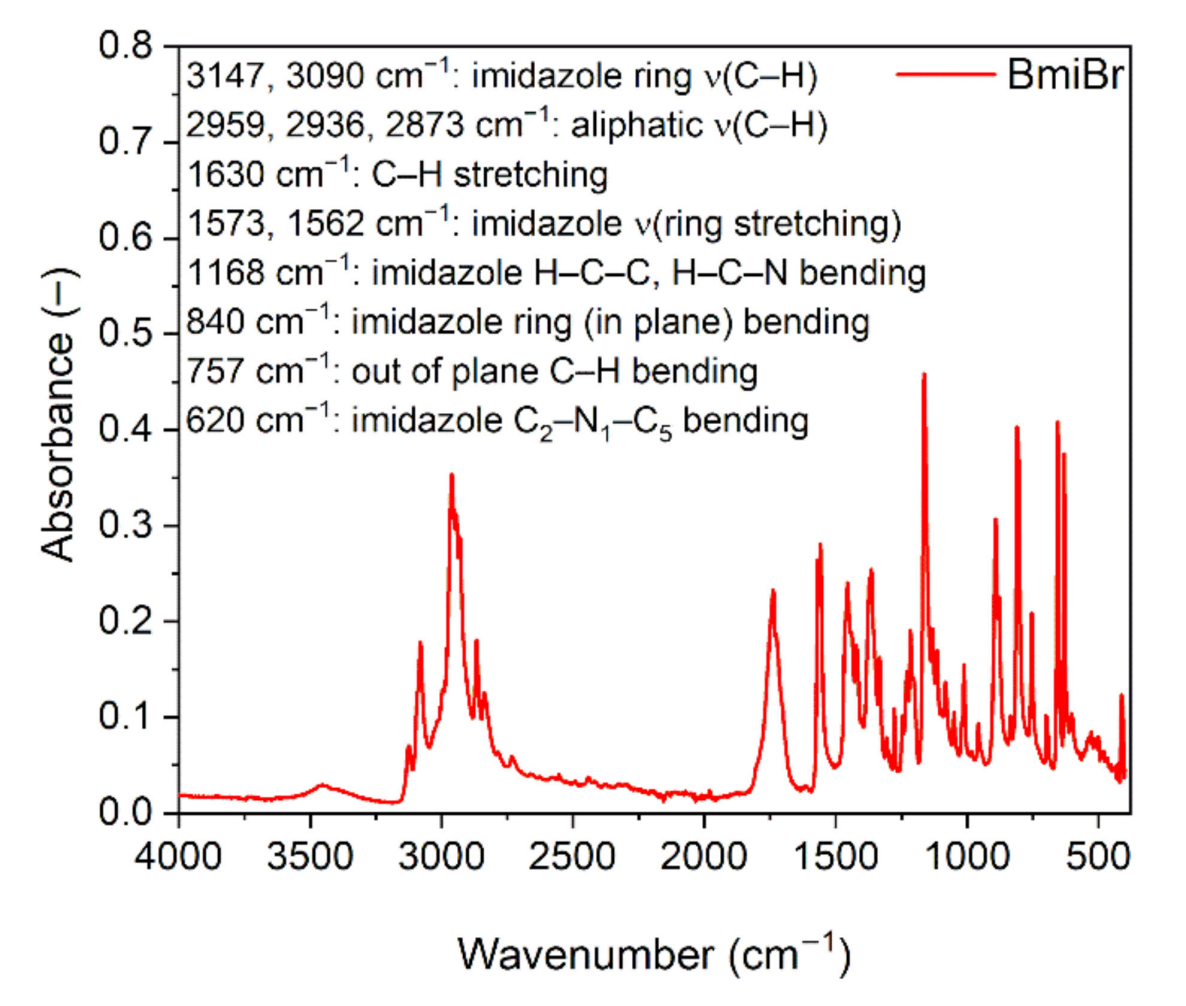
FT-IR spectrum of pure BmiBr.

**Figure 12:**
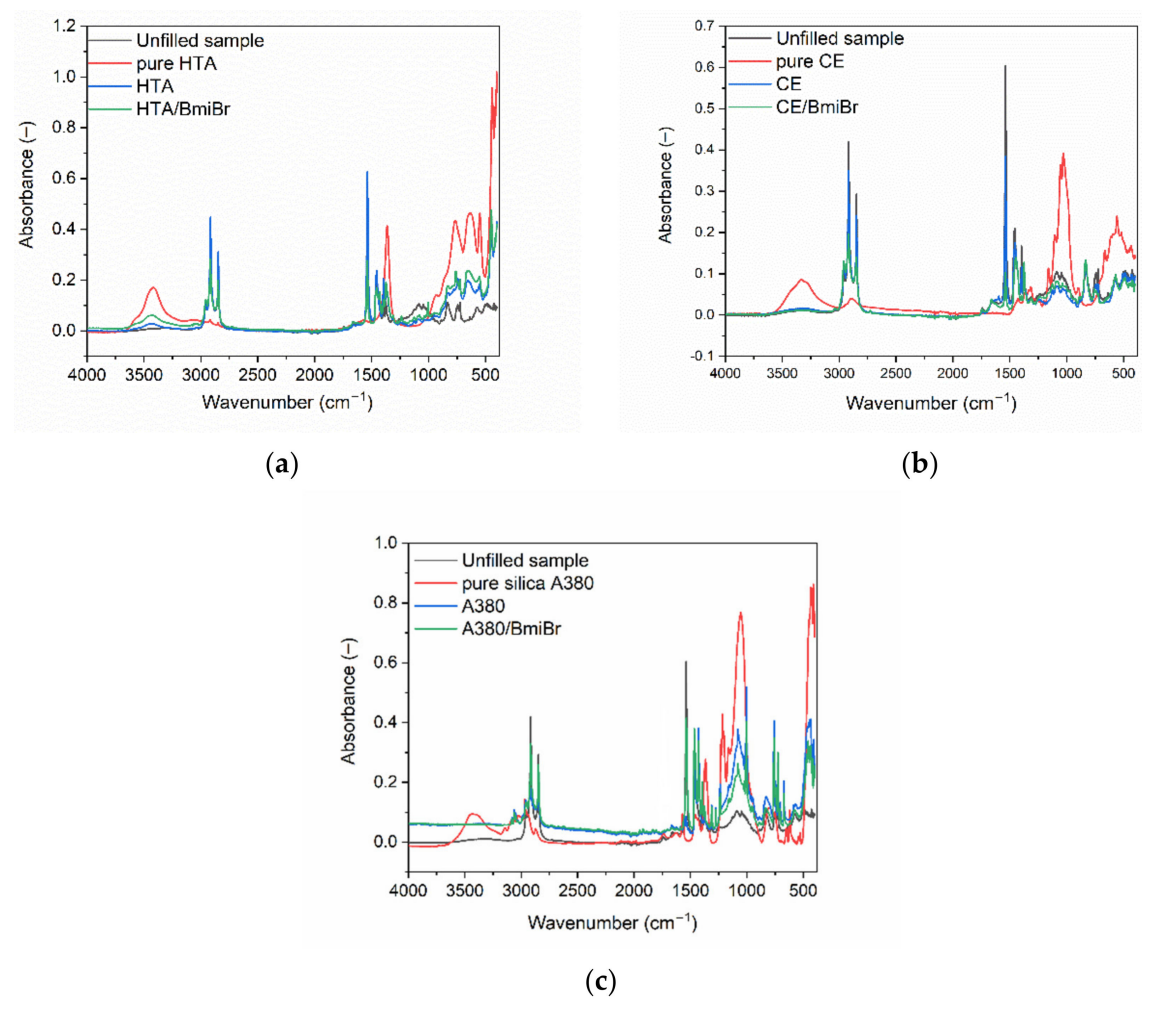
FT-IR spectra for NR vulcanizates filled with: (**a**) HTA; (**b**) CE; (**c**) A380.

FTIR spectroscopy was employed to investigate the possible interactions between NR, fillers and ILs. Measurements were carried out for pure fillers and ILs, as well as for NR vulcanizates. All the reported peak values were based on the maximum peak height of the unfitted spectra. Regarding the effect of ILs, the results for BmiBr were presented as an example, since no significant differences between the FT-IR spectrum of BmpyrBr and BmiBr were observed The FT-IR spectrum of BmiBr is presented in Figure 11, and is consistent with the FT-IR spectrum reported for this ionic liquid by other researchers [91,92]. The FT-IR spectra collected for NR vulcanizates are shown in Figure 12.

Analyzing the FT-IR spectrum of the pure BmiBr, some characteristic bands at 690–590, 1600–1080 and 3200–2800 cm^−1^ were obtained, which correspond to the C–H stretching vibrations of butyl chain and bands characteristic of the aromatic imidazolium ring [93,94,95,96]. In addition to these characteristic peaks, BmiBr is hydrophilic and shows the presence of some water at about 3450 cm^−1^ [91].

Regarding NR vulcanizates, the absorption bands of a strong amplitude characteristic for cis-1,4-polyisoprene were recorded at: 2961 cm^−^^1^ (–CH_3_, asymmetric stretching vibrations), 2970–2830 cm^−^^1^ (–CH_2_, symmetric stretching vibrations), 1647 cm^−^^1^ (C=C, stretching vibrations in NR compounds), 1494, 1432, 1394, 1375 cm^−^^1^ (–CH_2_, deformation vibrations). These bands are characteristic for the crosslinked NR composite and are similar to those described by other researchers [97,98]. Additionally, a broad peak at approximately 3200–3500 cm^−^^1^ was observed for HTA and CE-filled NR, which can be related to the presence of hydroxyl groups. This band was reported to be generated after addition of fillers, which have a hydrophilic character [97].

Analyzing the FTIR spectrum of NR compounds filled with HTA (Figure 12a), a broad band ranging from 3800 to 2500 cm^−1^ was detected, which is characteristic for O–H stretching of water molecules. Hydroxyl groups in the lamellae structure of LDHs and the band at 1650 cm^−1^ is also attributed to angular deformations of water molecules. Some bands related to metal–oxygen–metal vibrations were also found in the region of 1000–400 cm^−1^ [99]. Four bands were observed at 1363, 767, 626 and 549 cm^−1^, respectively, which correspond to the CO^2−^ group present in the structure of HTA. The intensity of these peaks was lower in the FT-IR spectrum of HTA-filled NR in comparison with pure HTA. A similar observation can be made for the intensity of the bands characteristic of OH groups. It can, therefore, be assumed that the aforementioned groups participate in the filler–filler and polymer–filler interactions and in the formation of a filler network in the crosslinked NR matrix. After the addition of BmiBr, no changes were observed in the FT-IR spectrum of NR vulcanizate apart from the bands characteristic of the imidazolium ring of the ionic liquid at 1168 and 840 cm^−1^.

According to the literature referring to FT-IR analysis of cellulose supramolecular structure, the most interesting bands are: 3700–3000 cm^−1^, associated with the hydrogen bond formation; 1420–1430 cm^−1^, attributed to the crystalline structure of the cellulose; between 900–890 cm^−1^, which correspond to the amorphous region of cellulose [100,101]. Analyzing the FT-IR spectrum for pure cellulose and for CE-filled NR vulcanizates (Figure 12b), some characteristic absorption bands were observed at the following wavenumbers: 3333 cm^−1^ and 1100 cm^−1^ (–OH, hydroxyl moieties), 2893 cm^−1^ (C–H, stretching vibrations), 1315 cm^−1^ (C–O, C=O, C=C, COOH), 1161 cm^−1^ (C–O, stretching, C–O–C, bridge vibrations), 1030 cm^−1^ (C–O, stretching vibrations), 1100–1000 cm^−1^ (CO–O–CO), 1200–900 cm^−1^ (–OH, –COO), 559 cm^−1^ (C–OH, out of plane bending, C–C). Similar results were also reported by other researchers [102,103]. Due to the incorporation of cellulose into NR matrix, some changes were observed in the FT-IR spectra of NR vulcanizates. For CE and CE/BmiBr samples, the bands in the spectral region of 1800–1500 cm^−1^ can be separated in two parts: between 1800–1610 cm^−1^ and between 1610–1500 cm^−1^ [104]. In the first part, a small intensity peak occurred, which corresponds to two vibrational modes at 1650 cm^−1^ and 1664 cm^−1^, respectively, attributed only to the C=C stretching vibration of the NR matrix. The second part corresponds to the –OH bending modes, owing to the cellulose. High-intensity bands collected in the range of 2970–2848 cm^−1^, 1540–1370 cm^−1^ and 835 cm^−1^ for NR vulcanizates filled with CE (with and without the addition of BmiBr) may indicate significant interactions between the NR matrix and cellulose, as postulated in the literature [104].

Analyzing FT-IR spectrum of pure silica A380 (Figure 12c), a broad band in the wavenumber range of 3500–2800 cm^−1^ was detected, with different contributions at 3438 and 2969 cm^−1^. Owing to the hydrophilic character of A380, the broad, intense peak at 3438 cm^−1^ occurred, which showed a water contribution. This was also ascribed to Si–OH stretching vibrations of surface silanol groups. Overtone modes of silica frame occurred between 2100 and 1800 cm^−1^ [105]. Additionally, the band at 1571 cm^−1^ was achieved due to the vibrations of physically adsorbed water. The peaks at 1365, 1228, 1217 and 1054 cm^−1^ were assigned to Si–O stretching vibrations. The band at 792 cm^−1^ corresponds to the O–H bending mode of hydrogen bonded. Vibrations occurring at 433, 426, and 421 cm^−1^ were characteristic of oxygen motions, both in plane Si–O–Si bending vibrations and out of plane rocking motions. In the FT-IR spectra of A380-filled vulcanizates (with and without BmiBr), some differences were observed compared to the spectra of unfilled NR and pure silica A380. As was expected, the characteristic silica bands appeared in the spectra of the filled vulcanizates as compared to the spectrum of unfilled NR. The intensity of bands in the range of 1250–800 cm^−1^, as well as at 792 cm^−1^ and 764 cm^−1^, corresponding to the Si–O and O–H vibrations, were decreased compared to the spectra of pure silica, which may indicate interactions between NR matrix and the filler. The spectrum of BmiBr-containing vulcanizate was similar to that of the vulcanizate without BmiBr and no significant changes were observed in the intensity or position of the bands. It should be noticed that vulcanized rubber is a medium of high viscosity and, in addition to the filler–polymer or filler–filler interactions, the bands visible in the FT-IR spectrum and their positions are also influenced by the bonds formed in the crosslinking reactions. Hence, the observation of the interactions between the filler and the elastomer matrix using FT-IR spectroscopy is very difficult, especially in the crosslinked elastomer matrix. These interactions could more easily be observed in NR latex.

## 4. Conclusions

In this work, the influence of different fillers, i.e., hydrotalcite, cellulose and silica, as well as ionic liquids with bromide anion and different cations, on the properties of NR composites were examined. The obtained results proved that the type of filler and the structure of ILs affected the rheometric properties and cure characteristics of NR compounds, as well as the performance of the NR vulcanizates.

Silica A380 considerably prolonged the scorch time and optimal vulcanization time of NR compounds, owing to the adsorption of curatives onto the silica surface, reduced the activity of the crosslinking system. Moreover, silica A380 significantly increased the viscosity of the uncrosslinked rubber compound compared to the unfilled NR. The applied fillers, especially silica A380, increased the maximum torque during vulcanization due to the introduction of the rigid phase of the filler into the soft elastomer matrix. ILs beneficially affected the cure characteristics of NR compounds, especially those filled with silica. The application of ILs reduced the viscosity of the uncrosslinked rubber compounds, which facilitated their processing, and enhanced the torque increase during vulcanization, owing to the improvement in the elastomer crosslinking degree. Moreover, the significant reduction in the optimal vulcanization time of NR compounds was achieved. The beneficial influence of ILs on the cure characteristics of A380-filled NR compounds resulted from the adsorption of ILs on the silica surface, which limited its ability to adsorb the curatives, and consequently improved the efficiency of crosslinking.

The type of filler and the structure of ILs affected the tensile properties of NR vulcanizates, but the influence of filler type was much more pronounced. The HTA-filled vulcanizates exhibited a higher tensile strength than the unfilled rubber, whereas vulcanizates containing CE and silica showed a significantly lower TS. The beneficial effect of HTA on the tensile strength resulted from both the reinforcing effect of HTA and the highest crosslink density of the HTA-filled vulcanizates. Regardless of the filler used, the addition of ILs, especially BmiBr, increased the tensile strength of the NR vulcanizates due to an improvement in their crosslink density.

Regardless of the filler and ILs used, NR vulcanizates exhibited approximately 200–300% lower elongation at break than the unfilled vulcanizate. This resulted from both the increase in the stiffness of the material due to the introduction of a filler into the elastomer matrix and the increase in the crosslink density of the vulcanizates compared to the unfilled one. All tested fillers enhanced the hardness of NR vulcanizates compared to the unfilled rubber. The most pronounced influence on the hardness was seen in silica A380. ILs increased the hardness of the vulcanizates containing silica and HTA but had no significant influence on the hardness of the CE-filled elastomer.

Owing to the reduction in the elasticity of NR vulcanizates, the applied fillers, particularly silica A380, decreased the mechanical loss factor at the glass transition temperature. Regardless of the filler used, ILs did not significantly alter the loss factor in the measured temperature range, and consequently had no considerable influence on the damping properties of the NR vulcanizates.

The type of filler and ILs greatly affected the onset decomposition temperature of the vulcanizates. CE and silica A380 improved the thermal stability of the NR vulcanizates without ILs. The A380-filled vulcanizate exhibited a significantly higher thermal stability than the unfilled one, due to both the high thermal stability of silica itself and the network formed by the silica nanoparticles dispersed in the elastomer matrix, which hindered the diffusion of gases and volatile products of thermal decomposition through the elastomer. On the other hand, HTA-filled vulcanizate had a considerably lower thermal stability compared to the unfilled sample, which resulted from thermal decomposition of HTA, which started at a temperature above 150 °C. Regardless of the filler used, ILs deteriorated the thermal stability of NR vulcanizates. However, despite the reduction in the onset decomposition temperature, the NR vulcanizates were still thermally stable up to a temperature of approximately 300 °C.

The presented results show that the FT-IR technique might be successfully employed to analyze the structure of pure fillers, pure ILs or unfilled NR. However, such an analysis is founded only on data generation and their proper fitting. Finally, the obtained results indicate the appropriate functional groups, which can be assigned to proper chemical compounds. However, to estimate possible interactions between the components of NR composites, further analysis with other methods, e.g., solid-state nuclear magnetic resonance (CMR) or time-of-flight secondary ion spectrometry (TOF-SIMS), would be more helpful.

## Figures and Tables

**Table 1:** Ionic liquids (ILs) with bromide anion used in natural rubber (NR) compounds.

Name	Abbreviation	CAS Number	Purity (%)	Water Content (wt.%)
1-butyl-3-methylimidazolium bromide	BmiBr	85100-77-2	≥99.0%	<0.5%
1-butyl-1-methylpyrrolidinium bromide	BmpyrBr	93457-69-3	≥99.0%	≤0.5%

**Table 2:** General recipes of natural rubber (NR) reference compounds, parts per hundred of rubber (phr); (MBT, 2-mercaptobenzothiazole).

Ingredient, Phr	Unfilled Sample (R0)	NR/A380 (R1)	NR/HTA (R2)	NR/CE (R3)
NR	100	100	100	100
Sulfur	2	2	2	2
ZnO	5	5	5	5
MBT	2	2	2	2
Stearin	1	1	1	1
Silica	₋	30	₋	₋
Hydrotalcite	₋	₋	30	₋
Cellulose	₋	₋	₋	30

**Table 3:** General recipes of NR compounds containing ionic liquids (phr); (BmiBr, 1-butyl-3-methylimidazolium bromide; BmpyrBr, 1-butyl-1-methylpyrrolidinium bromide).

Ingredient, Phr	NR/BmiBr (IL1–IL3)	NR/BmpyrBr (IL4–IL6)
NR	100	100
Sulfur	2	2
ZnO	5	5
MBT	2	2
Stearin	1	1
Filler *	30	30
BmiBr	3	₋
BmpyrBr	₋	3

* Silica, hydrotalcite or cellulose, alternatively.

**Table 4:** Thermal stability of pure fillers (T_5%_, decomposition temperature at 5% of the mass change; T_DTG_, DTG peak temperature; standard deviations (SD): T_5%_, T_DTG_ ± 3 °C).

Filler	T_DTG_ (°C)	Mass Losses (%)
Hydrotalcite	240	Δm_25–250 °C_: 13.9
	445	∆m_250–800 °C_: 31.3
Cellulose	70	∆m_25–150 °C_: 10.1
	365	∆m_150–500 °C_: 86.5
	630	∆m_550–800 °C_: 3.8
Silica A380	70	∆m_25–800 °C_: 6.4

**Table 5:** Cure characteristics at 160 °C of NR compounds (S_min_, minimum torque; S_max_, maximum torque; ∆S, torque increase; t_02_, scorch time; t_90_, optimal vulcanization time; standard deviations: S_min_ ± 0.1 dNm, S_max_ ± 1.3 dNm, ΔS ± 1.5 dNm, t_02_ ± 0.1 min, t_90_ ± 0.4 min).

Compounds	S_min_ (MPa)	S_max_ (MPa)	∆S (MPa)	t_02_ (min)	t_90_ (min)
Unfilled sample	0.3	5.3	5.0	0.9	2.2
BmiBr	0.2	6.0	6.2	0.7	1.7
BmpyrBr	0.2	5.6	5.8	0.8	1.9
Hydrotalcite					
HTA	0.2	7.5	7.3	0.7	1.8
HTA/BmiBr	0.2	9.1	8.9	0.6	1.6
HTA/BmpyrBr	0.2	8.1	7.9	0.6	2.1
Cellulose					
CE	0.2	7.6	7.4	0.8	2.2
CE/BmiBr	0.2	8.3	8.1	0.7	2.2
CE/BmpyrBr	0.2	7.3	7.1	0.7	2.4
Silica A380					
A380	7.7	12.9	5.2	3.9	15.9
A380/BmiBr	5.7	17.8	12.1	0.5	2.3
A380/BmpyrBr	6.1	17.2	11.1	0.6	2.4

**Table 6:** Differential scanning calorimetry (DSC) analysis of NR compounds (T_g_, glass transition temperature; ∆C_p_, heat capacity; ∆H, enthalpy of crosslinking; T_g_ ± 1 °C, ∆C_p_ ± 0.1 J/g × K, temperature ± 9.0 °C; ΔH ± 1.2 J/g).

NR Compounds	T_g_ (°C)	∆C_p_ (J/g × K)	Temperature of Vulcanization (°C)	∆H (J/g)
Unfilled sample	−63.8	0.43	147–215	6.4
Hydrotalcite				
HTA	−64.6	0.34	126–213	14.7
HTA/BmiBr	−63.9	0.32	128–235	18.7
HTA/BmpyrBr	−64.1	0.35	126–237	13.9
Cellulose				
CE	−63.2	0.33	129–232	12.0
CE/BmiBr	−64.1	0.35	126–238	16.6
CE/BmpyrBr	−64.8	0.36	128–232	13.0
Silica A380				
A380	−63.9	0.31	149–232	12.0
A380/BmiBr	−64.2	0.32	146–219	10.9
A380/BmpyrBr	−64.4	0.35	149–216	12.7

**Table 7:** Mechanical properties and hardness of NR vulcanizates (SE_300_, stress at 300% relative elongation; TS, tensile strength; EB, elongation at break; H, hardness).

NR Vulcanizates	SE_300_ (MPa)	TS (MPa)	EB (%)	H (ShA)
Unfilled sample	2.5 ± 0.4	17.4 ± 1.0	830 ± 24	30 ± 1
Hydrotalcite				
HTA	3.4 ± 0.3	20.1 ± 0.6	623 ± 18	37 ± 1
HTA/BmiBr	6.1 ± 0.1	23.8 ± 1.6	527 ± 16	41 ± 1
HTA/BmpyrBr	5.0 ± 0.2	21.7 ± 1.4	601 ± 14	42 ± 1
Cellulose				
CE	1.6 ± 0.3	9.1 ± 0.6	629 ± 18	42 ± 1
CE/BmiBr	2.6 ± 0.1	10.7 ± 0.2	578 ± 4	43 ± 1
CE/BmpyrBr	2.5 ± 0.1	10.2 ± 0.5	610 ± 16	44 ± 1
Silica A380				
A380	3.2 ± 0.1	8.7 ± 0.2	634 ± 21	49 ± 1
A380/BmiBr	4.4 ± 0.1	13.4 ± 0.9	599 ± 16	52 ± 1
A380/BmpyrBr	4.6 ± 0.3	11.7 ± 0.5	526 ± 18	52 ± 1

**Table 8:** Glass transition temperature (T_g_) determined by dynamic mechanical analysis (DMA) and mechanical loss factor (tan δ) of NR vulcanizates (SD: T_g_ ± 1.6 °C; Tan δ_Tg_ ± 0.2; Tan δ_25–50 °C_ ± 0.02).

NR Vulcanizates	T_g_ (°C)	Tan δ_Tg_ (-)	Tan δ_25 °C_ (-)	Tan δ_50 °C_ (-)
Unfilled sample	−68.9	2.5	0.04	0.04
Hydrotalcite				
HTA	−68.2	2.2	0.03	0.03
HTA/BmiBr	−71.1	2.1	0.03	0.03
HTA/BmpyrBr	−71.5	2.0	0.03	0.03
Cellulose				
CE	−67.8	1.8	0.04	0.05
CE/BmiBr	−68.9	1.7	0.05	0.04
CE/BmpyrBr	−69.0	1.8	0.04	0.05
Silica A380				
A380	−70.9	1.0	0.10	0.10
A380/BmiBr	−69.8	1.0	0.09	0.10
A380/BmpyrBr	−71.5	1.0	0.10	0.10

**Table 9:** Onset temperature of thermal decomposition (T_5%_), temperature of the DTG peak (T_DTG_), and total mass loss (∆m) during decomposition of NR vulcanizates (SD: T_5%_ ± 1.3 °C; T_DTG_ ± 1.2 °C; ∆m ± 1.3 %).

NR Vulcanizates	T_5%_ (°C)	T_DTG_ (°C)	∆m_25–600 °C_ (%)	∆m_600–800 °C_ (%)	Residue at 800 °C (%)
Unfilled sample	310	402	94.7	1.3	4.0
Hydrotalcite					
HTA	284	396	83.5	0.9	15.6
HTA/BmiBr	271	396	83.5	1.8	14.7
HTA/BmpyrBr	263	400	83.0	1.8	15.2
Cellulose					
CE	317	398	94.8	2.1	3.1
CE/BmiBr	294	399	93.7	3.2	3.1
CE/BmpyrBr	288	399	92.7	3.3	4.0
Silica A380					
A380	335	401	74.5	1.6	23.9
A380/BmiBr	323	400	75.6	2.4	22.0
A380/BmpyrBr	320	398	75.5	2.3	22.2

## Data Availability

The data presented in this study are available on request from the corresponding author.
